# Supplementary material for: Vitamin D Modified DSS-Induced Colitis in Mice via STING Signaling Pathway
Source: Biology (Basel). 2025 Jun 18;14(6):715. doi: 10.3390/biology14060715 (PMC12190092; doi:10.3390/biology14060715)
Supplement: Supplementary file 1 [file biology-14-00715-s001.zip › Supplementary Materials S1: 16s-report of gut microbiota/Groups/diff_analysis/heatmap/GBMlogdiffheatmap.pdf]

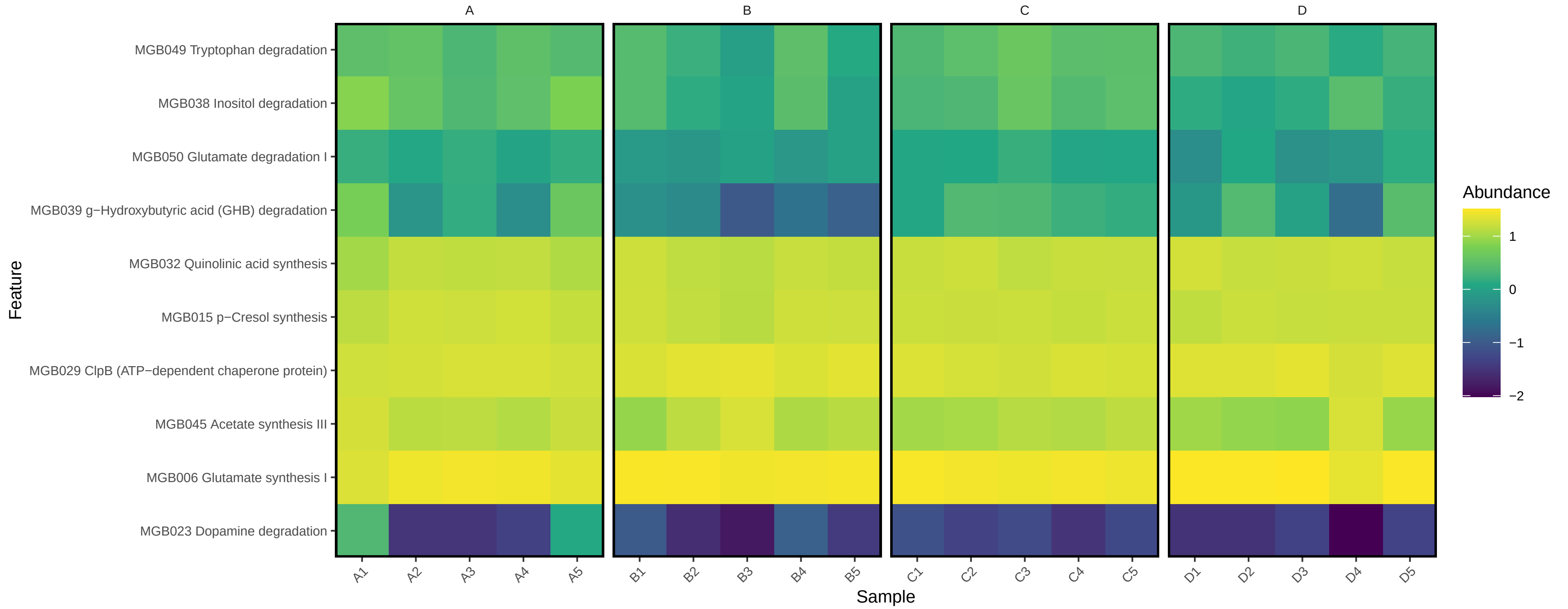

A
B
C
D
MGB049 Tryptophan degradation
MGB038 Inositol degradation
MGB050 Glutamate degradation I
MGB039 g−Hydroxybutyric acid (GHB) degradation
MGB032 Quinolinic acid synthesis
MGB015 p−Cresol synthesis
MGB029 ClpB (ATP−dependent chaperone protein)
MGB045 Acetate synthesis III
MGB006 Glutamate synthesis I
MGB023 Dopamine degradation
Feature
A1 A2 A3 A4 A5 B1 B2 B3 B4 B5 C1 C2 C3 C4 C5 D1 D2 D3 D4 D5
Sample
Abundance
1
0
−1
−2
